# Mr. Melhuish, on the Influenza

**Published:** 1803-10-01

**Authors:** Thomas Beddoes


### *To the Editors of the Medical and Physical Journal.*

GENTLEMEN,

I Send you a few more Reports. I had good reason to expect information from remote parts of Ireland, which to me would have been exceedingly interesting. But public events will account either for delay or disappointment.

There is one request which I would beg leave to address to your Correspondents in general. This is to add to their other communications, of whatever nature these may be, the date of the rise, progress, and termination of the influenza, whenever they know it with tolerable certainty.

It is hardly necessary to state how a knowledge of its first appearance bears upon the most interesting question that can be started respecting this complaint. I shall have occasion to point out this at large, in a communication I mean to offer you for insertion next month.

I am, &c.

*September* 7, 1803. THOMAS BEDDOES.

---

83. Dr. DAVIES, Carmarthen, July 21, 1803.

The influenza appeared at Carmarthen, and the neighbourhood, about the latter end of February, and continued until about the middle of May.

Although it appeared general, yet many, very many, escaped it.

It did not appear to me to be contagious. In my own family it clearly was not so, nor do I consider that it was so in this part of the country.

Its attack commenced with violent head-ach, pain in the limbs, a prostration of strength, pain in the chest, with cough, very violent in some instances, and the expectoration often streaked with blood. Hæmorrhagies from the nose were not unfrequent. In some cases a violent ear-ach, and in many, abscesses formed in the ear. In two patients, ulcers of a very fœtid kind appeared in the mouth.

The pulse was quick, the thirst great, and the respiration in some remarkably oppressive.

I did not observe any peculiar foulness in the tongue.

Delirium often attended these symptoms, especially in old people. In one case (a gentleman of 76) the delirium continued six or seven days; I considered it to be the effect of debility. I found

I found in this complaint the cascarilla bark of infinite service. The Peruvian bark, certainly, did not appear to me generally to agree. I gave the cascarilla bark after the second or third day, and with unquestionably good effects.

Where the liver was previously affected, the recovery was uncommonly slow, and lingering. In one or two instances ascites followed.

In all the observations that I made, (which embraced a practice of nearly fifty miles) the effect of the influenza appeared to be extreme debility, and the recovery from it in a most remarkable manner slow and tedious.

In a gentleman of sixty-five, paralytic symptoms showed themselves, and the whole terminated in a mortification of the feet, and death. The mortification of the feet shewed itself about a month from the first attack. The patient was of a thin spare habit, and in good health before the influenza attacked him. The paralytic symptoms appeared chiefly to affect the muscles, the throat, &c. This is the only fatal case that came within my knowledge.

I treated the coughs in their usual manner with blisters, &c. always having in view the support of the general strength. I did not bleed in any case.

84. Mr. Melhuish, Tiverton, August 18, 1803.

The influenza first appeared in two of my patients, one of which resided in this town, and the other about a mile south-east of Tiverton, on the 23d day of March last.

This disease, after having been very general about a *month*, subsided almost immediately after the change of the wind from the east (in which quarter it had generally been from the time abovementioned) to the west, and the subsequent rain, without recurring in any single instances.

It appeared to me as uniformly endemic and sometimes epidemic.

I recollect the former influenza some years since, which seemed a compound disease of catarrh and peripneumony, the first rather predominating; in the late disease (in my opinion) a compound disease also of peripneumonia notha, catarrh, and sometimes inflammatory sore throat; the peripneumonia notha was most predominant. An intermittent tendency was also *very* observable in some instances, but it never continued so as to require the use of the bark.

I found the pneumonic affection in this disease infinitely less dangerous than when the peripneumonia notha is less frequent, for though three instances in this neighbourhood occurred where two in each house died, yet these were old persons, persons, or of very bad stamina, and in a bad state to meet the influenza. Upon the whole, I found it *much* less dangerous than from the severity of the symptoms I had first apprehended.

I had only two cases in which I bled, in one of which the patient was accustomed to bleeding every spring; the other highly plethoric; but each recovered.

I treated the disease exactly as peripneumonia notha, except that opium at night, which I have so often given with the greatest success in this disease, unfortunately had generally a contrary effect in the influenza.

85 Dr. Grant, Waterford, July 20, 1803.

As I kept no diary during the prevalence of the influenza, I send what I can recollect.

It was very general here from the beginning of April till the middle of May, but though you could meet few in the street who did not complain of it, as if it were the fashion, very few were so ill as to require medical attendance, nor did I learn that any one died of it here.

It was not contagious, as in several families where I attended not more than one or two were affected. It ceased, as well as I remember, when the weather became *settled* warm.

It was the same through the country round.

The symptoms I met were shivering more or less, a sense of tightness and oppression of the breast, a small cough, and sometimes failure of the voice, a thin discharge from the nose and eyes with great complaint of weariness, particularly of the loins; the treatment I used was antiphlogistic regimen, keeping the belly free with neutral salts.

86. Dr. Ryan, Kilkenny, July 28, 1803.

The first time I was called upon to visit a person ill of the influenza was on the 9th or 10th of April, and my conversation with the gentleman took place the day after. As you know I have already alleged that it was in consequence of this conversation, I pronounced my first patient to be ill of the epidemic, and I can positively assert I proclaimed its existence in this town at a time when no idea whatever was entertained of such a troublesome visitor having got footing amongst us; however, I will not take upon myself to declare that this was in fact the first case that appeared.

Relapses were so frequent, and the reliques of the influenza so completely resembled the original, that it is extremely difficult to ascertain the precise period of its complete extinction, though I think I can speak with confidence as to the time it began to take flight. I was particularly careful to insert the day of the month I commenced and ceased my attendance on every person of consideration during the prevalence of this disorder; and from this registry it appears, that about the latter end of May, or the beginning of June, its violence so completely subsided as no longer to excite any interest or alarm.

I enclose you Dr. Grant's letter, upon which it is necessary however to remark, that the intercourse between this town and Waterford is by an indirect and circuitous way, while that between Cork and Dublin is direct and constant, a mail-coach from each of these cities arriving here every day in the week, so that supposing the disorder to be infectious, and to prevail equally in the three sea-port towns at the same time, it is extremely improbable indeed that the contagion could be received from Waterford so early as from Cork or Dublin.

The features and character of the epidemic did not appear to me to be influenced by the weather, nor was there any striking difference to be observed between the influenza which was to be met with in the town and that in very distant country places. Individuals indeed on recovery suffered severely at times from north-east winds, by exposing themselves too soon and too incautiously to the open air.

### 87. Dr. Kinglake, Taunton, August 6, 1803.

The first instance which occurred in my observation of the late influenza, was about the middle of February; the last, at the beginning of June, though it is reported to have had both an earlier and later existence in this vicinity.

Its prevalence in different places was not simultaneous, it proceeded desultorily in some cases, in others it was more regularly progressive.

After it had attained its height, or greatest frequency, it continued to shew itself in single instances during several weeks, more particularly towards its termination.

Well marked examples often presented of its personal communication. It occurred to me often to remark, that when the disease had been unduly protracted, and had proved unusually violent, that those who were most occupied pied in the care of the patient, successively suffered. This fact was so striking, that it soon confirmed me in the propriety of analogically extending to the disease, my *general persuasion* of all morbid action being more or less contagious, according to the degree of the malady, personal proximity, existing temperature, and temperamental susceptibility for diseased impression.

The influenzal disorder (as in every other instance of epidemic affection) probably had its source in the noxious influence of variable temperature, deranging the due distribution of the circulating fluids, vitiating the various secretions, and distempering the healthful conditions of vital motion, from whence generally resulted inflammatory determination to the mucous membrane of the trachea, bronchiæ, fauces, and nostrils, which became sympathetically diffused over the system, in the form of febrile affection.

The disease appeared to have had this catarrhal origin, and generally the catarrhal form. In some few cases its pressure was concentered on the intestines, when it exhibited the strict character of enteritis; in others, bilious suffusion gave it an icteric complexion.

It was purely inflammatory; it speedily, and under my direction, almost invariably yielded to reduced temparature, administered by atmospheric exposure, copious dilution with cold water, and the rigid avoidance of dietetic, medicinal, and mental stimulants.

The want of universality in its personal contagion is no valid objection to its infectious nature. A variety of circumstances must concur to give effect to contagious power. Adequate virulence, suitable distance, and above all, an apt state, or susceptibility for morbid impression, must present, to render the communicable quality operative.

No greater difficulty occurs in explaining the numerous exceptions and anomalies, in the agency of personal contagion, than in those of atmospheric infection; in both, they are equally referable to concomitant circumstances, which widely modify the influence, but do not annul the specific power.

In short, my opinion is, that the late influenza (in common with every variety of epidemic disease, not excepting even the plague, originated from the morbid influence of variable temperature, by which the salutary conditions of vital motion became at length so distempered, as to genarate and evolve from every part of the stystem an *halitus*, or *materies morbi*, proving infectious or not, according to its degree of concentration, and the existing temperamental aptitude for being impressed by it. In many instances it may be efficient, while in others it may be quite inert. A similar operation of cause and effect, obtains in every description of disease that disturbs vital motion sufficiently to vitiate its salutary procesess or functions.

The principle of contagion, consisting of a specific arrangement of matter, issues from every conceivable disease, but it amounts to operative force in comparatively few instances.

It may be fairly questioned, if the atmospheric air is susceptible of any condition but that of temperature, that could be generally active in the production of disease. Neither its mechanical impregnation with noxiously stimulant substances, whether in the form of miasma or animalcula, nor any chemical combination of its constituent principles in unnatural and deleterious proportions, could be admitted as efficient causes of epidemic diseases, consistently with the original mildness, gradual aggravation, limited extension, and personal communication of the morbid influence.

These hurtful agents must necessarily be too insulated for general diffusion, nor could their universal influence be compatible with animal existence.

Various remote causes may engender individual distempers, but these will not become general or epidemic in the multifarious dissimilitude of temperamental susceptibility, without the agency of specific matter, combined and combinable only by the peculiar affinities of *animal chemistry*. It does not appear that the late influenza was distinctly communicable by clothes or fomites. Its contagious quality did not seem to be sufficiently powerful to impart diseased impression, but when applied in the full force of direct personal transference.

88. Mr. Bartley, Nailsworth, Gloucestershire, August 26, 1803.

In regard to your queries respecting the influenza, I will endeavour to afford you all the information on the subject within my power of communication, which I fear is but little. It commenced here in the beginning of March, about which time I attended a gentleman who was, I believe, the first in this neighbourhood who was attacked; a very short time afterwards, I became myself the subject of the disease; the symptoms of which I will briefly recount as they occurred to me. I first experienced a very unpleasant pleasant and painful sensation, as though gravel or some other extraneous matter was introduced between the globe of the eye and eye-lid. Considerable redness in the eye, impatience of light, and a profusion of tears soon followed. This was shortly succeeded by a great discharge of mucus from the nose, of so ichorous a quality as to excoriate the parts over which it passed, accompanied with violent pain in the head, slight cough, chilliness alternating with heat, pain in the back, and oppression at the præcordia. Appetite very much impaired, and debility excessively great. Every symptom increased in the evening; sleep was constantly interrupted; and during the two or three first nights, my imagination was so confused and bewildered as almost to approach delirium. Bowels costive; urine high coloured, small in quantity, and from its heat occasioning much pain in the discharge. After applying a vesicatory inter scapulas, the inflammation of the eyes subsided, and in about four days the febrile symptoms disappeared, but I experienced for some time afterward increasing debility, and an inconceiveable, insurmountable dejection of spirits, with frequent nausea, and great disinclination to food. Becoming at length convalescent, I attended several persons in the same complaint, and soon after suffered a relapse nearly as violent as the first attack, save that the inflammation returned in the left eye only, which I relieved by keeping up for some time a discharge from a blister on the adjoining temple. Whether, in the first instance, my disorder proceeded from epidemical influence of the atmosphere alone, or not, I will not pretend to determine; but in the second, I am persuaded it was the effect of contagion, and was by that means communicated to every person composing my household; and I have constantly observed, that in houses where the disease made its appearance, all the family have in turn been the subjects of it, and other families, who avoided communication with the sick, wholly escaped the disease.

It may be useful to remark, that in no two persons I have observed precisely the same symptoms. Some, in addition to those I have enumerated, were troubled with a *violent* cough and excessive pain at the sternum, which commenced in the incipient stage of the disorder, in which cases the disease terminated with a profuse expectoration of high coloured yellow mucus, not frothy, but resembling pus in its appearance, and of a disagreeable odour, "sui generis." In others, it commenced with vomiting; every thing taken into the stomach was speedily rejected, which in general relieved the head, abated the febrile symptoms, and induced a quicker termination of the disease. In others, there appeared symptoms of pneumonic inflammation. In others, cynanche tonsillaris. Many were affected with obstinate costiveness, whilst others laboured under profuse diarrhœa. The pulse in general was frequent, but not hard. The tongue, in many instances, white in the middle, but red round the edges, and covered with florid papillæ; but in some cases it exhibited no unusual appearance. The disease in general terminated about the fourth or fifth day, but in aged and infirm patients, it was much protracted; the debility was consequently greater and of longer continuance. I have found the disease fatal in one instance only, where it was succeeded by hydrothorax, which has terminated in death. It disappeared in general here about the latter end of April, but there were a few solitary instances of its re-appearance in remote situations, and, as I think, communicated by fomites.

I shall venture still farther to intrude on your patience by relating my plan of treatment, which, of course, varied as much as the symptoms. In the commencement of the disease, I have generally exhibited (particularly where nausea has existed, in which case a vomit was usually previously administered) a saline mixture in a state of effervescence, with a few drops of vin. antim. tartaris. or aq. ammon. acet. with mist. camphor. which, together with a strict observance of an antiphlogistic regimen, contributed to abate the pain in the head, and also the febrile symptoms. To obviate costiveness, cooling laxatives were administered occasionally, such as infus. tamarindorum cum senna, &c. I did not attempt to check diarrhœa where it appeared during the height of fever, but when that subsided, a small dose of tinct. opii has usually moderated the discharge. Where there appeared to be pneumonic inflammation, I did not hazard the use of the lancet on account of excessive debility. A vesicatory applied to the affected part, constantly relieved the pain, and produced the desired effect. I did not venture on the use of opiates until the disappearance of fever, when a little tinct. opii camph. with lac. ammoniaci and oxymel scillæ has been useful in allaying the cough and promoting expectoration. As the great subsequent prostration of strength indicated the use of corroborants, I exhibited the tonic bitters and steel with much advantage, and in some cases where cough was not present tinct. cinchon. comp.

I have, I doubt not, been unnecessarily prolix on the subject, subject; nor have I replied to your queries in the order they are proposed, for this I solicit your excuse, as I have not at present, time for revision. You will be able to perceive at least from what I have stated, that I am decidedly of opinion, that the disease may be communicated by infection or fomites, and that it essentially differs from common catarrh. If the incoherent observations I have made can afford you the least possible satisfaction, it will abundantly gratify me.

89. Mr. Hugo, Crediton, August 10, 1803.

The first appearance of the disorder in this neighbourhood which came under my observation, was on the 22d of March, in the family of a gentleman who resides about three miles *west* of this town. He had been attending, with his lady, the assize at Exeter, the whole of the preceding week, at which time the influenza was very general there. They came home both ill of the disease. On the next day, the servant who returned with them, was seized with it, and by the 25th, it had been communicated to every other person in the house. Some labourers, who reside at an adjoining farm, were affected about the same time; but a woman who had been employed at Exeter, was the first attacked by it. It appeared very soon afterwards in the town of Crediton, and here also the first case I visited was a gentleman who had been attending at the assize. It spread very rapidly, and in a short time became general in the town and adjoining villages; its duration as an epidemic, was from the 22d of March to the end of April; it was afterwards only occasionally met with.

The symptoms and mode of treatment were so similar to those which are commonly described, that it is unnecessary to enumerate them. Very few fatal cases occurred, and they were all persons in advanced life, where the disease was accompanied with peripneumony of the low typhoid kind.

From the manner of its commencement and progress here, I can scarcely doubt its infectious nature, and believe it to have been brought into this town by persons who received the contagion at Exeter. It is scarcely credible that any miasmata floating in the atmosphere, can be carried in a direction different from the current of air existing at that particular time, which was far from being the case in this instance, or that it can be conveyed to a distant situation without producing its effects also on some part of the intermediate country; yet the last place in this neighbourhood which was visited by the influenza, is a village situate at least six miles *eastward* of the house where I first observed it. It appears also that though the contagion on a large scale proceeded, in this part of the kingdom, from east to west, when considered with respect to particular situations, its course was by no means so regular, but seemed to depend rather on the degree of intercourse which subsisted between the uninfected and those who were ill of the disorder. In fact, I consider it as an epidemic, spreading in the same manner, and proceeding in the same course, as those diseases which are indisputably contagious.

The principal reason for referring it to an infected atmosphere, seems to arise from the circumstance of a large number of persons being seized so nearly at the same time; but if we reflect that the disorder when mild so much resembles a common catarrh that the patient does not submit to confinement, but mixes unreservedly with his neighbours, and also that the febrile action probably takes place within a short time after the contagion is received, there will be little difficulty in reconciling it to the same laws on which every other infectious disease is communicated and depends.

